# Clinical Presentation of Preeclampsia and the Diagnostic Value of Proteins and Their Methylation Products as Biomarkers in Pregnant Women with Preeclampsia and Their Newborns

**DOI:** 10.1155/2018/2632637

**Published:** 2018-06-28

**Authors:** Maria Portelli, Byron Baron

**Affiliations:** Centre for Molecular Medicine and Biobanking, Faculty of Medicine and Surgery, University of Malta, Msida MSD2080, Malta

## Abstract

Preeclampsia (PE) is a disorder which affects 1-10% of pregnant women worldwide. It is characterised by hypertension and proteinuria in the later stages of gestation and can lead to maternal and perinatal morbidity and mortality. Other than the delivery of the foetus and the removal of the placenta, to date there are no therapeutic approaches to treat or prevent PE. It is thus only possible to reduce PE-related mortality through early detection, careful monitoring, and treatment of the symptoms. For these reasons the search for noninvasive, blood-borne, or urinary biochemical markers that could be used for the screening, presymptomatic diagnosis, and prediction of the development of PE is of great urgency. So far, a number of biomarkers have been proposed for predicting PE, based on pathophysiological observations, but these have mostly proven to be unreliable and inconsistent between different studies. The clinical presentation of PE and data gathered for the biochemical markers placental growth factor (PlGF), soluble Feline McDonough Sarcoma- (fms-) like tyrosine kinase-1 (sFlt-1), asymmetric dimethylarginine (ADMA), and methyl-lysine is being reviewed with the aim of providing both a clinical and biochemical understanding of how these biomarkers might assist in the diagnosis of PE or indicate its severity.

## 1. Introduction

Preeclampsia (PE) is a multisystem, pregnancy-specific disorder that is characterised by the development of hypertension and proteinuria (elevated levels of protein in the urine) after 20 weeks of gestation [1]. PE is a leading cause of maternal, perinatal (from the 20th week of gestation to the 4th week after birth), and foetal/neonatal mortality and morbidity worldwide [2, 3].

PE is a very significant disease which complicates from 2% to 5% of pregnancies in Europe and America and can reach up to 10% of pregnancies in developing countries, mainly due to the lack of or inadequacy of emergency care [2]. Also, PE is associated with an increased risk of placental abruption, preterm birth, foetal intrauterine growth restriction (IUGR), acute renal failure, cerebrovascular and cardiovascular complications, disseminated intravascular coagulation, and maternal death [4]. Therefore, the ability to provide an early diagnosis of PE is vital.

## 2. Clinical Presentation, Diagnosis, and Pathophysiology of PE

Clinically, PE presents as new-onset hypertension in a previously normotensive woman, with systolic and diastolic blood pressure readings of ≥140 and ≥90 mmHg, respectively, on 2 separate occasions that are at least 6 hours apart, together with proteinuria that develops after 20 weeks of gestation [5–7].

This disorder can have an early onset (PE starting before 34 weeks of gestation) or late onset (after 34 weeks of gestation) and can be classified as mild or severe, depending on the severity of the symptoms present [2] (Table 1). In the case of severe PE, more significant blood pressure elevations and a greater degree of proteinuria are noted. Other symptomatic features of severe PE which may be present include oliguria (less than 500 mL of urine in 24 hours), cerebral or visual disturbances, and pulmonary oedema or cyanosis [8, 9].

Also, the clinical presentation of PE may be either insidious or fulminant since some women may be asymptomatic initially, even after hypertension and proteinuria are noted, while others may present symptoms of severe PE from the start [1]. Finally, this condition may present itself as a maternal disorder only, such that there is normal foetal growth, or else it may lead to intrauterine growth restriction or sudden foetal distress [2].

**Figure 1:**
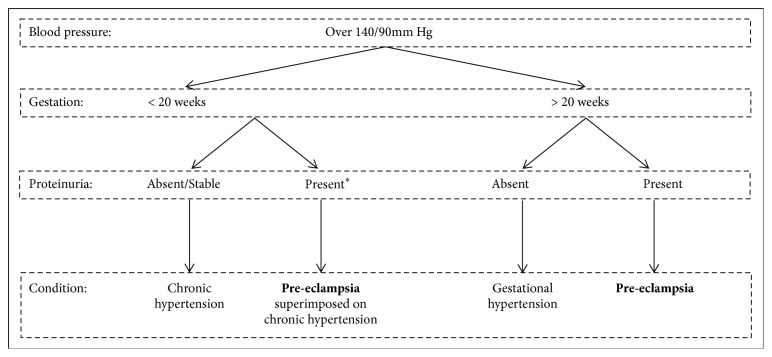
Simplified diagnostic information for the distinction of different types of hypertension and preeclampsia. *∗*In the form of new or increased proteinuria, together with development of increasing blood pressure, or HELLP syndrome [9].

Hypertensive disorders of pregnancy are the most common complications seen by obstetricians [10] and they are all associated with higher rates of maternal and foetal mortality and morbidity [11]. This category of disorders includes chronic hypertension, PE, PE superimposed on chronic hypertension, and gestational hypertension [9]. The aetiologies and pathology of these disorders vary, and thus obtaining a diagnosis of PE becomes less difficult if physicians are able to differentiate PE from the other hypertensive disorders of pregnancy (Figure 1).

In chronic hypertension, the elevated blood pressure may predate the pregnancy, be noted before 20 weeks of gestation, or else be present 12 weeks after delivery [9]. This contrasts with PE, which is defined by the presence of elevated blood pressure and proteinuria after 20 weeks of gestation. In severe cases, PE can evolve into eclampsia which is a severe complication that is characterised by new-onset of epileptic seizures (generalised convulsions), due to angiospasms in the brain and brain oedema [13], in a woman with PE [1]. Eclampsia usually occurs in the second half of pregnancy and is a significant cause of maternal death, most commonly as a result of cerebral haemorrhage [14].

PE superimposed on chronic hypertension is characterised by new-onset proteinuria (or by a sudden increase in the protein level if proteinuria was already present), an acute increase in blood pressure (assuming proteinuria already exists), or the development of the HELLP (haemolysis, elevated liver enzyme, low platelet count) syndrome [8]. Finally, gestational hypertension can be distinguished from PE since it is characterised by the presence of elevated blood pressure after 20 weeks of gestation, which normalises within 12 weeks after delivery, together with the absence of proteinuria [8].

**Figure 2:**
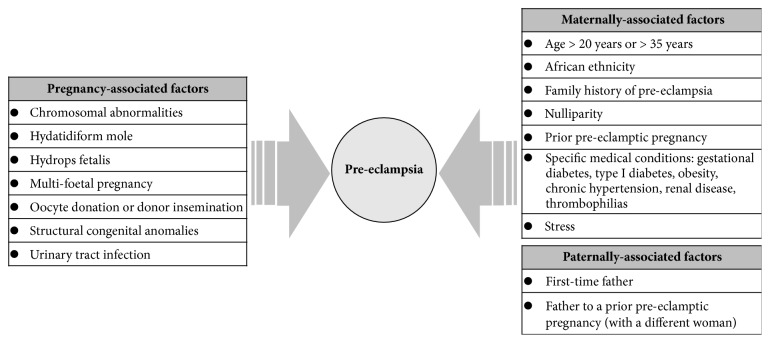
Risk factors for PE associated with the pregnancy itself or with specific parental characteristics from both maternal and paternal side [8, 12].

Medical conditions which have a potential to cause microvascular disease, including diabetes mellitus, chronic hypertension, and vascular and connective tissue disorders, as well as antiphospholipid syndrome and nephropathy, are all risk factors for developing PE. A number of other risk factors for developing PE, which can be associated with the pregnancy itself or with the clinical characteristics of the mother or father of the foetus, are presented in Figure 2 [8, 12, 15].

Although the pathophysiology of PE is not fully understood, problems of placental implantation and the level of trophoblastic invasion, as a consequence of endothelial dysfunction, appear to play a central role in the development and progression of this disorder. During normal pregnancy, cytotrophoblasts derived from the foetus invade and remodel the maternal uterine spiral arteries such that these small diameter, high-resistance arteries are converted into high capacity, low-resistance vessels [16]. This process is completed around midgestation in order to optimise the distribution of the maternal blood and ensure that the developing uteroplacental unit has adequate oxygen and nutrient delivery from the maternal circulation (Ramsey and Donner (1980) as cited in [3]).

PE is thought to evolve in two stages. The first, asymptomatic stage of PE involves impaired trophoblastic invasion of the decidua (maternal placental bed) that seems to be due to local, abnormal foetomaternal immune interactions within the uterine wall [2, 17, 18]. This abnormal, shallow placentation reduces uteroplacental blood perfusion and consequently leads to local placental hypoxia. This oxidative stress has been shown to further aggravate vascular function in the placenta [19], which consequently leads to insufficient blood perfusion, inflammation, apoptosis, and structural damage [17, 20–23].

In the second stage, placental blood-borne factors released into the maternal circulation from the poorly perfused placenta, together with the aberrant expression of proinflammatory, antiangiogenic, and angiogenic factors, may activate the maternal endothelium and will eventually cause the endothelial dysfunction that leads to the main clinical symptoms of PE: hypertension and proteinuria [14, 24]. It has been noted that the magnitude of defective trophoblastic invasion of the spiral arteries correlates with the severity of PE [25].

Although PE is not preventable, PE-related mortality can be decreased through early detection and careful monitoring of PE [1]. Also, women who have progressive or severe PE should be hospitalised early on to allow close monitoring of both the maternal and foetal health condition.

## 3. Biomarkers of PE

The search for noninvasive, blood-borne, or urinary biomarkers that could be used to screen for and diagnose this life-threatening disorder of pregnancy is of utmost importance. Such biomarkers could predict the development of PE or assist in its detection, which in turn could have a vital impact on the management of pregnant women and their unborn children [2].

Most significantly, screening pregnant women with the use of biochemical markers for PE could enable presymptomatic diagnosis which will in turn reduce unnecessary suffering and healthcare costs associated with this disorder [26]. By providing an earlier diagnosis, progression of the disorder can be monitored more closely, together with the maternal and foetal health condition, thus allowing for more optimised time for delivery with the aim of reducing the number of premature births or other complications associated with PE [27]. Such biochemical markers may also allow the categorisation of women with PE according to the severity of the symptoms and/or pregnancy outcome which would further improve their clinical management [28].

Numerous biochemical markers for PE, which were selected based on pathophysiological observations noted in cases of PE, such as placental and/or endothelial dysfunction, have been investigated (Table 2). However, the reliability of these markers in predicting PE has been inconsistent between different studies [2]. Consequently, this review will focus on those biochemical markers for PE which appear to be most clinically relevant, alone or in combination, for the diagnosis of PE as well as in their ability to give an indication of the severity of this disorder, namely, placental growth factor (PlGF), soluble Feline McDonough Sarcoma- (fms-) like tyrosine kinase-1 (sFlt-1), and asymmetric dimethylarginine (ADMA), as well as introducing the possibility of screening for methyl-lysine in pregnancy-related proteins.

### 3.1. Placental Growth Factor (PlGF) and Soluble fms-Like Tyrosine Kinase-1 (sFlt-1)

PlGF belongs to the vascular endothelial growth factor (VEGF) family of proteins and it shares 53% identity with the platelet-derived growth factor-like region of VEGF [29]. Based on this homology with VEGF, PlGF was proposed to be an angiogenic factor [29–31]. In fact, PlGF was seen to possess strong angiogenic and mitogenic properties which are capable of inducing the proliferation, migration, and activation of endothelial cells [32, 33].

The expression of PlGF messenger RNA (mRNA) appears to be restricted to the placenta, trophoblastic tumours, and cultured human endothelial cells [29–31]. Essentially, PlGF is found in high amounts in the placenta, but it is also expressed at a low level under normal physiological conditions in several other organs including heart, lung, skeletal muscle, and adipose tissue [34–40].

The proangiogenic activity of members of the VEGF family of proteins, including PlGF, is achieved through the binding and activation of tyrosine kinase receptors [41, 42]. The most important receptors, which were found to bind the VEGF family of proteins with high affinity, are the fms-like tyrosine kinase receptor (Flt-1, also referred to as VEGF receptor 1, VEGFR1) and kinase domain region (KDR or VEGFR2) [43, 44]. These receptors are made up of a single signal sequence, a transmembrane domain, 7 immunoglobulin-like domains in their extracellular domain (the ligand-binding domain), and an intracellular tyrosine kinase domain [45].

However, it was noted that a cDNA in the endothelial cells of the human umbilical vein in the placenta encodes a truncated form of Flt-1 which is generated through alternative splicing of the mRNA. This soluble isoform of Flt-1 (sFlt-1) lacks the seventh immunoglobulin-like domain, the cytoplasmic domain, and the transmembrane sequence [45].

#### 3.1.1. PlGF and sFlt-1 in Disease States and Pregnancy

One of the most important properties of vascular endothelial cells is their ability to proliferate and form a network of capillaries through a process termed angiogenesis [33]. In a normal adult, the angiogenic process is tightly regulated and is limited to the endometrium and the ovary during the different phases of the menstrual cycle, and to the heart and skeletal muscles following injury due to prolonged and sustained physical exercise [134]. This process is especially prominent during embryonic development (Ramsey & Donner (1980) and Gilbert (1988) as cited in [33]) since angiogenesis is essential for correct development of the embryo and for postnatal growth [134].

The complex interplay between some members of the VEGF family of proteins, including PlGF, and their cognate receptors, especially Flt-1, is essential for angiogenesis to occur [2]. On the other hand, the soluble splice variant of Flt-1, sFlt-1, is secreted into the circulation and acts as an antiangiogenic factor since it antagonises and neutralises PlGF and VEGF by binding to them and inhibiting their interaction with endothelial receptors on the cell surface [36, 45, 57].

PlGF is present during early embryonic development and throughout all the stages of pregnancy since it is highly expressed by the placenta. It has been suggested that the presence of this proangiogenic factor serves as a control for trophoblast growth and differentiation [31, 37], which in turn implies that PlGF has a role in the invasion of the trophoblast into the maternal decidua [135]. Concurrently, although sFlt-1 is secreted in small amounts by endothelial cells and monocytes, the placenta seems to serve as the major source of sFlt-1 in the circulation during pregnancy. This finding is emphasised by the significant fall in the level of circulating sFlt-1 following the delivery of the placenta [47].

**Figure 3:**
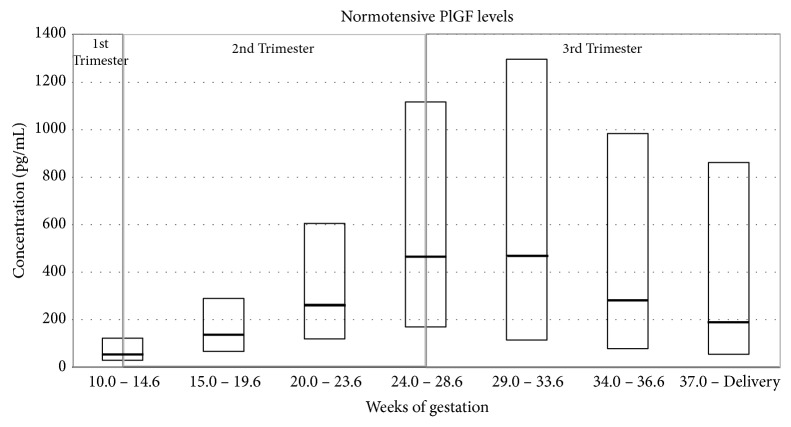
PlGF levels (pg/mL) measured in normotensive women during different weeks of gestation (based on data from Roche Study no. CIM RD000556/X06P006).

**Figure 4:**
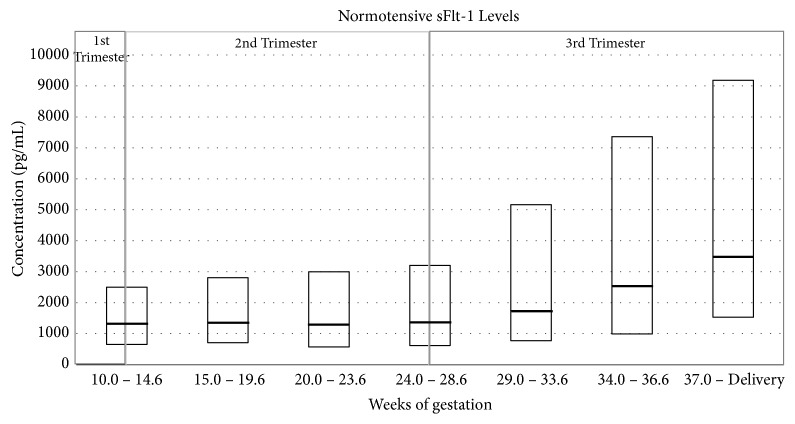
sFlt-1 levels (pg/mL) measured in normotensive women during different weeks of gestation (based on data from Roche Study no. CIM RD000556/X06P006).

In a normotensive pregnant woman, the level of PlGF in the maternal circulation increases gradually during the first two trimesters and peaks at midgestation, before declining again as the pregnancy comes to term. Alternatively, the sFlt-1 level in normotensive pregnant women remains relatively stable during the first two trimesters, after which it increases steadily until term [45, 47, 53]. This gestational variation can be observed in the results presented in the charts below which were obtained in the Prospective Multicenter Study: Diagnosis of Preeclampsia (Roche Study no. CIM RD000556/X06P006). In this study, the PlGF and sFlt-1 levels were measured in normotensive women from countries across Europe, who had singleton pregnancies and went on to have normal pregnancy outcomes (no PE/HELLP and no IUGR) (Figures 3 and 4).

The levels of these biomarkers have also been investigated in the maternal circulation of patients with PE. There is strong evidence for the reduced occurrence of free, bioactive PlGF, together with higher placental expression of sFlt-1 and, consequently, elevated levels of circulating sFlt-1 in preeclamptic patients during active disease when compared with normotensive pregnant women [46, 47, 50, 53, 55, 67].

In a large cross-sectional study comparing gestational age-matched women with active PE and normotensive pregnancies, PlGF levels were noted to be lower and sFlt-1 levels to be higher in the preeclamptic group (mean PlGF level of 137pg/mL versus 669pg/mL; mean sFlt-1 level of 4382pg/mL versus 1643pg/mL) [53]. The decrease in PlGF levels is thought to be due to the increased concentration of circulating sFlt-1 from 33 to 36 weeks of gestation and hence increased binding of PlGF to sFlt-1, rather than the decrease in PlGF caused by reduced production of PlGF [53].

In 2003, Maynard et al. introduced exogenous sFlt-1 into pregnant rats, and remarkably this led to reduced levels of PlGF, hypertension, and proteinuria, symptoms parallel to those observed in patients with PE [47]. This finding led to the idea that the maternal endothelial dysfunction that is noted in preeclamptic patients is caused by the imbalance of the levels of pro- and antiangiogenic factors in the maternal circulation. There is much supportive evidence suggesting that the antagonism of PlGF by sFlt-1 may be responsible for the endothelial dysfunction in PE [59, 60].

The PlGF deficiency and sFlt-1 excess observed in preeclamptic patients may also be due to the placental hypoxia that is associated with incomplete remodelling of the maternal spiral arteries. This defective placentation, as a result of incompletely remodelled arteries, offers persistently high resistance to uterine artery blood flow, which may in turn predispose to vascular rupture in the placental bed, especially after the onset of hypertension [136, 137]. However, more evidence is required to determine whether the altered levels of these pro- and antiangiogenic factors are the consequence or the cause of the placentation defect in women with PE.

**Figure 5:**
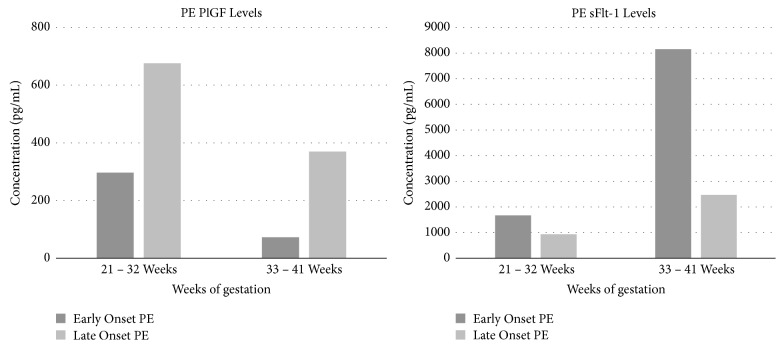
PlGF and sFlt-1 results obtained in women with early onset and late onset PE during different gestational periods [53].

**Figure 6:**
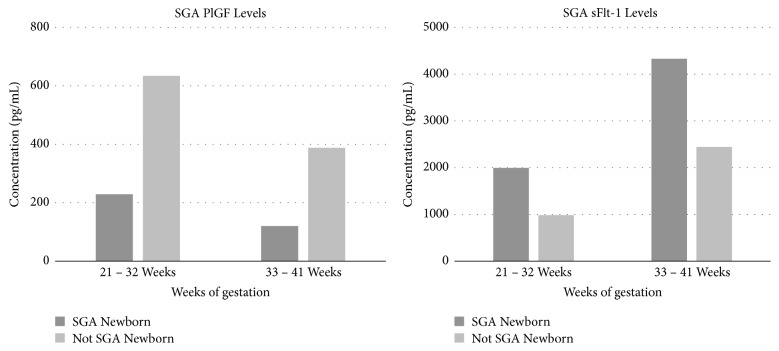
PlGF and sFlt-1 results obtained during different gestational periods in women with PE who later delivered small for gestational age (SGA) newborns and those that delivered infants of normal gestational size [53].

Studies have shown that the level of maternal PlGF was more significantly reduced in patients with severe symptoms of PE compared to normotensive pregnant women and women with symptoms of mild PE. On the other hand, in the case of maternal sFlt-1 levels, the increased levels were shown to correlate with the severity of PE, with mean sFlt-1 levels ranging from 1.50 ± 0.22ng/mL in normotensive pregnant women to 3.28 ± 0.83ng/mL in women with mild PE and to 7.64 ± 1.5ng/mL in women with severe PE [47, 48]. Furthermore, it has been noted that the variation in PlGF and sFlt-1 is more pronounced in early onset PE when compared to late onset PE as well as in women who had PE and later delivered small for gestational age (SGA) newborns [53, 56]. The results obtained by Levine et al. [53] which show these differences are presented in Figures 5 and 6.

In the study by Levine et al., it was also reported that the increase in sFlt-1 levels in the circulation of patients with PE corresponds to a decrease in free PlGF [53]. Moreover, it was also observed that the alterations in the levels of these factors precede the clinical diagnosis by several weeks. In fact, a significant finding in this study was that the elevated level of sFlt-1 can be detected in the maternal serum 5 weeks before the clinical symptoms of PE appear while the decreased PlGF level can be detected from 13 to 16 weeks of gestation in women who subsequently develop PE. This finding was later observed by a number of other studies [20, 53, 58, 62–66, 68–72, 138].

**Figure 7:**
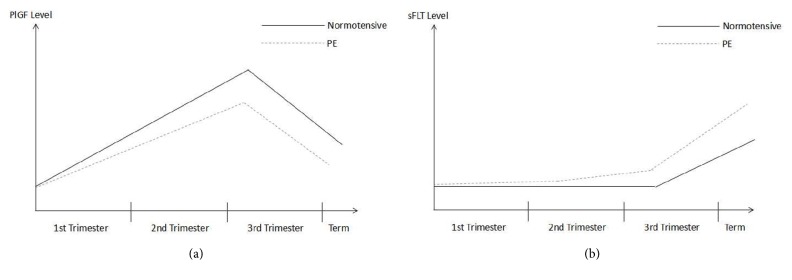
Levels of (a) PlGF and (b) sFlt throughout normotensive pregnancy as compared to levels in preeclamptic pregnant women.

These findings have suggested that the measurement of PlGF and sFlt-1 may be used to predict the development of PE several weeks before the clinical onset of symptoms of this disease (Figure 7). The combined measurement of PlGF and sFlt-1 also distinguished women who subsequently developed PE from women who subsequently developed gestational hypertension, delivered SGA newborns, or completed a normal, healthy full term pregnancy [47, 52].

According to some studies, altered levels of sFlt-1 are specific for PE since no changes are detected in women who subsequently delivered SGA newborns or whose pregnancies were complicated by IUGR when compared to normotensive women with normal pregnancy outcomes [51, 58]. However, in a selected group of patients with abnormal uterine perfusion with subsequent IUGR, other studies have detected similar alterations in PlGF and sFlt-1 levels during the second trimester [139].

**Figure 8:**
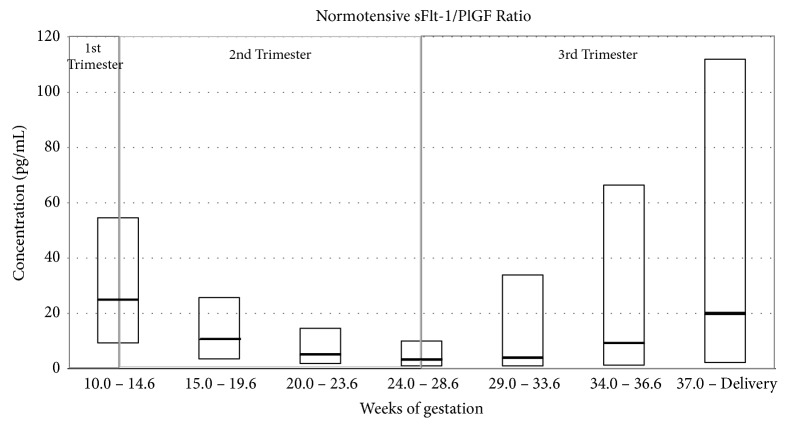
PlGF to sFlt-1 ratio (pg/mL) measured in normotensive women during different weeks of gestation (based on data from Roche Study no. CIM RD000556/X06P006).

The combination of sFlt-1 and PlGF values in the form of a ratio, as shown for the Prospective Multicenter Study: Diagnosis of Preeclampsia (Roche Study no. CIM RD000556/X06P006) (Figure 8), has also been used as a predictor of PE. In a prospective study by Rana et al., [61] it was suggested that the ratio of sFlt-1 to PlGF appears to be a better predictor of PE than either measure alone. Kim et al. [24] revealed that the sFlt-1 to PlGF ratio in preeclamptic women was significantly higher when compared to the normal controls since the median value for the log [sFlt-1/PlGF] ratio in preeclamptic women was 1.6 (range 1.0 – 2.9), while the median value in the normotensive controls was 1.2 (range 0.5 – 1.9). In this study, a cut-off value of 1.4 was used since this showed 80.4% sensitivity and 78% specificity, with women having maternal log [sFlt-1/PlGF] ratio values more than 1.4 being at a higher risk of developing PE. Therefore, this ratio is a reliable marker of overall risk of PE and it may be used to distinguish between normal pregnancy and pregnancy complicated by PE and to define the severity of PE [2, 140].

The measurement of PlGF and sFlt-1 has only rarely been extended to the infants born from preeclamptic pregnancies. In 2005, Staff et al. measured PlGF and sFlt-1 levels in normotensive and preeclamptic pregnant women and their newborns [49]. The results obtained for the mothers reflected the same results obtained by studies mentioned in previous sections, with lower PlGF and higher sFlt-1 levels being noted in the preeclamptic group. In this study, the umbilical samples obtained from all newborns had PlGF levels that were below the concentration of the lowest standard of the ELISA kit used in the study (15.6pg/mL) and thus comparison between the preeclamptic and normotensive control groups could not be achieved. On the other hand, the median sFlt-1 concentration obtained for foetuses born to mothers with PE was found to be significantly higher than the median concentration obtained for those born to normotensive mothers (246 pg/mL, 95% CI for the median 163–255 versus 163 pg/mL, 95% CI for the median 136–201).

At the same time, although sFlt-1 levels were noted to be higher in foetuses born to mothers with PE, the sFlt-1 concentrations measured in umbilical samples were noted to be very low when compared to the maternal sFlt-1 concentrations. This finding suggests that the foetus does not contribute significantly to the elevated maternal sFlt-1 concentration in PE, which further reinforces the assumption that the increase in circulating sFlt-1 concentration in mothers with PE originates primarily from the placenta [49]. This finding is also consistent with the idea that foetuses do not experience hypertension or proteinuria like their preeclamptic mothers because they are not exposed to high concentrations of antiangiogenic factors, including sFlt-1, which, although of placental origin, should be primarily restricted to the maternal vasculature [141].

### 3.2. Protein Methylation Products

**Figure 9:**
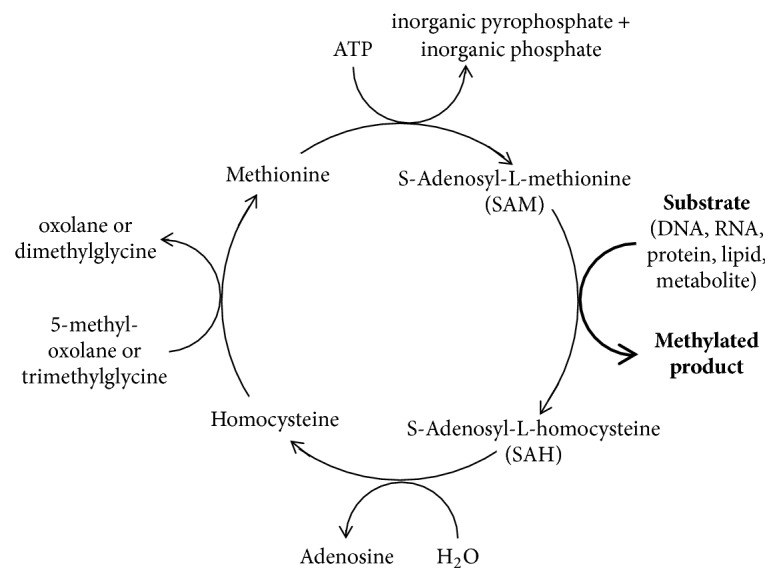
The S-adenosyl methionine cycle.

Protein methylation is a posttranslational modification (PTM) that involves the transfer of methyl groups from S-adenosyl-L-methionine (SAM) to a particular protein residue under the control of specific methyltransferase enzymes [142]. This results in the generation of a methylated substrate and the by-product, S-adenosyl-L-homocysteine (SAH), which is then degraded by the enzyme S-adenosylhomocysteine hydrolase to give adenosine and homocysteine [143] (Figure 9). Such PTMs predominantly target the side chains of arginine and lysine, but other amino acid residues, including histidine, asparagine, glutamine, and cysteine, have been shown to serve as minor targets for methylation.

#### 3.2.1. Asymmetric Dimethylarginine (ADMA)

**Figure 10:**
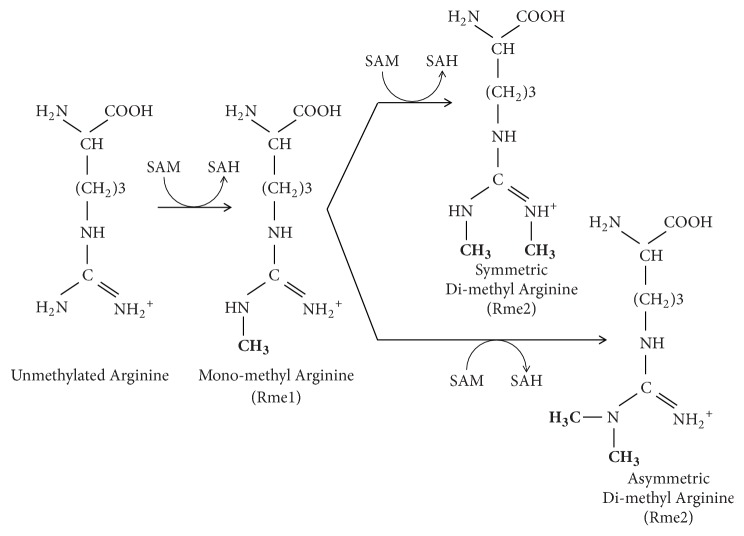
Formation of mono-, symmetrical, and asymmetrical dimethylarginine.

Different types of methylarginine are synthesised following arginine methylation, which is a PTM of the nitrogen atom forming part of the guanidino moiety of the arginine (R) group within proteins. Proteins that undergo arginine methylation are involved in a number of different cellular processes, including transcriptional regulation, RNA metabolism, and DNA damage repair [144]. This process involves the addition of one or two methyl groups, derived from S-adenosylmethionine (SAM) [145], to the guanidino nitrogen atom of arginine and is achieved with the help of protein arginine N-methyltransferase enzymes (PRMTs) which belong to a sequence-related family of methyltransferases [146]. The guanidino group of arginine can be methylated in three different ways to give *ω*-NG-monomethylarginine (MMA), *ω*-NG,N'G-symmetric dimethylarginine (SDMA), or *ω*-NG,NG-asymmetric dimethylarginine (ADMA) [144] (Figure 10).

**Figure 11:**
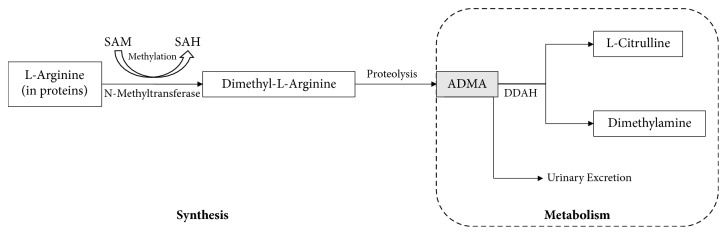
Overview of the synthesis and metabolism of ADMA. Synthesis of ADMA involves the methylation of arginine residues with the help of N-methyltransferase (protein arginine N-methyltransferases, PRMTs) which converts the methyl donor S-adenosylmethionine (SAM) to S-adenosylhomocysteine (SAH) followed by proteolytic breakdown of the proteins, which generates ADMA and N-monomethyl-L-arginine (L-NMMA). Elimination of ADMA is partly achieved via urinary excretion. However, ADMA is mainly eliminated through its metabolism to citrulline and dimethylamine by the enzyme dimethylarginine dimethylaminohydrolase (DDAH) [145].

ADMA is eliminated in part by urinary excretion, but it is mainly metabolised via hydrolytic degradation to citrulline and dimethylamine. This metabolic reaction is catalysed by the enzyme NG-dimethylarginine dimethylaminohydrolase (DDAH) [147] (Figure 11). There are 2 isoforms of DDAH: DDAH-1 and DDAH-2. Tissues expressing neuronal nitric oxide synthase (NOS) usually contain DDAH-1, while tissues containing the endothelial isoform of NOS (eNOS) predominantly contain DDAH-2 [148]. Thus, it has been observed that DDAH-1 is found in high levels in the kidneys and liver, whereas DDAH-2 is the most abundant isoform in the endothelium [145].

*(1) ADMA in Disease States and Pregnancy*. In 1992, it was reported that ADMA is an endogenous competitive inhibitor of NOS [149]. NOS is responsible for the synthesis of nitric oxide in endothelial cells since it catalyses the conversion of L-arginine to L-citrulline and NO [150]. ADMA is an analogue of L-arginine which is also synthesised and released by endothelial cells.

NO plays multiple roles in the cardiovascular system [144]. It is a potent vasoactive mediator that is released in response to stress [151] and is important in maintaining endothelial homeostasis [145]. Apart from inducing vasodilatation to regulate vascular tone and tissue blood flow [150–152], endothelial NO also inhibits platelet aggregation [153], inhibits adhesion of leukocytes and monocytes to the endothelium [154], and inhibits smooth muscle cell proliferation [155].

It has been noted that decreased levels or inhibition of DDAH, which is the enzyme that catalyses the hydrolysis of ADMA, results in higher levels of ADMA in the circulation and causes gradual vasoconstriction [156]. This occurs because the elevated level of ADMA in the circulation results in the reversible inhibition of endogenous NO synthesis which in turn could lead to endothelial dysfunction [157]. The low levels of NO result in increased systemic vascular resistance and blood pressure [75]. High levels of ADMA have been observed in individuals with cardiovascular diseases including atherosclerosis, hypertension, and hypercholesterolaemia and in individuals with chronic renal failure [145]. Conventional cardiovascular risk factors may reduce DDAH activity by increasing oxidative stress, and this will in turn also result in elevated levels of ADMA [148, 158–160].

**Figure 12:**
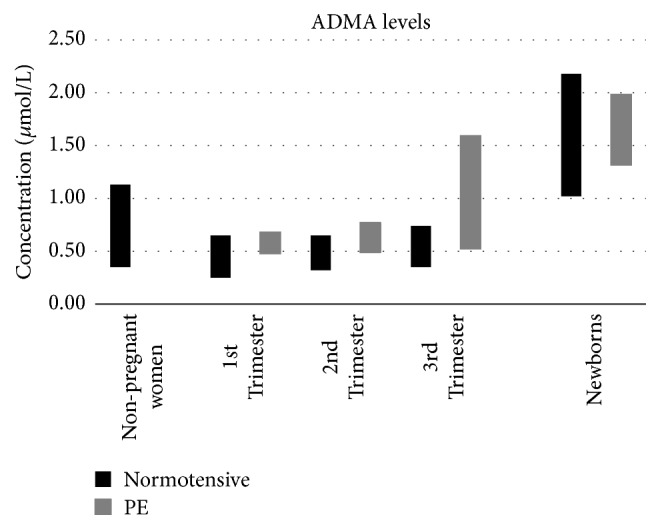
Levels of ADMA throughout pregnancy as compared to levels in nonpregnant women.

In a study in 1998 by Holden et al. [73], it was determined that pregnant women have a lower concentration of ADMA in their circulation than nonpregnant women. Their findings revealed that while the mean ADMA concentration in nonpregnant women was 0.82 ± 0.31*μ*mol/L, the mean values of ADMA in pregnant women were in the range of 0.40 ± 0.15 *μ*mol/L in the first trimester, 0.52 ± 0.20*μ*mol/L in the second trimester, and 0.56 ± 0.22*μ*mol/L in the third trimester. A similar observation was later made by Maeda et al. [79] who also noted lower mean ADMA concentrations in pregnant women (0.29 ± 0.05*μ*mol/L in the first and third trimesters and 0.32 ± 0.05*μ*mol/L at term) when compared to mean ADMA levels in nonpregnant women (0.41 ± 0.06*μ*mol/L). At the same time, the results obtained by Holden et al. revealed that although the mean ADMA levels are lower in pregnant women, these tend to increase during the normal gestational period [73]. This finding is not reflected in the results obtained by Maeda et al. since the latter group did not note a change in mean ADMA levels from the first to the third trimester (0.29 ± 0.05*μ*mol/L in the first and third trimesters), with the only increase being noted at full term (0.32 ± 0.05*μ*mol/L at term) [79]. Alternatively, the increase in mean ADMA levels during pregnancy was observed later on in a study by Rizos et al. in 2012 who showed that the mean ADMA levels in pregnant women increased from 0.51 ± 0.14*μ*mol/L in the first trimester to 0.52 ± 0.13*μ*mol/L in the second trimester and finally to 0.58 ± 0.16*μ*mol/L in the third trimester [81] (Figure 12). Such findings have suggested that ADMA may have a role in vascular dilation and blood pressure regulation during pregnancy [73].

Numerous studies have measured the level of ADMA in pregnant women to determine whether there is a significant difference in ADMA concentrations in the circulation of women with PE when compared to women with uncomplicated pregnancies.

Discrepant findings have been observed. In separate studies in 1998, both Holden et al. [73] and Pettersson et al. [80] observed elevated mean ADMA levels during the third trimester in preeclamptic patients (1.17 ± 0.42*μ*mol/L and 0.55 ± 0.02*μ*mol/L, respectively) when compared to the normotensive pregnant controls during the same gestational period (0.56 ± 0.22*μ*mol/L and 0.36 ± 0.01*μ*mol/L, respectively). Similarly, the study by Rizos et al. [81] also showed elevated mean ADMA levels during all three trimesters in preeclamptic patients (0.58 ± 0.10*μ*mol/L in the first trimester, 0.63 ± 0.14*μ*mol/L in the second trimester, and 0.68 ± 0.11*μ*mol/L in the third trimester) compared to women with uncomplicated pregnancies (0.51 ± 0.14*μ*mol/L in the first trimester, 0.52 ± 0.13*μ*mol/L in the second trimester, and 0.58 ± 0.16*μ*mol/L in the third trimester). However, in a number of other studies, although the median ADMA levels demonstrated a similar increased trend in preeclamptic patients, these findings were shown not to be statistically significant [74, 76, 77].

Furthermore, elevated ADMA concentrations have been noted in the circulation of pregnant women who went on to develop PE. This increased ADMA concentration was noted prior to the development of clinical signs and symptoms of PE [82, 83], which suggests that ADMA could have a role in the pathogenesis of this condition. Since nitric oxide is known to be important in maintaining both maternal and foetal blood flow and vascular tone and in maintaining the foetomaternal circulation, it has been proposed that elevated levels of ADMA in pregnancy, as well as the consequent decreased levels of NO in the circulation, may contribute to the pathophysiological features of PE [75, 79].

The measurement of ADMA levels in umbilical cord blood samples might be important to explain the regulatory mechanisms of the circulatory system during the perinatal period [84]. However, data regarding the level of ADMA in neonates is limited. In the previously mentioned study by Maeda et al. [79], it was also observed that the ADMA level measured in umbilical blood was significantly higher than the maternal level, which was noted to be highest at term (1.02 ± 0.18*μ*mol/L versus 0.32 ± 0.05*μ*mol/L, respectively). This finding was later observed by Tsukahara et al. [84], who noted that the ADMA levels measured in umbilical blood from control newborns (newborns born to normotensive mothers following uncomplicated pregnancies) were about two times higher than the ADMA levels measured in lactating women, healthy children, and healthy adults (1.71 ± 0.47*μ*mol/L versus 0.71 ± 0.06*μ*mol/L, 0.71 ± 0.11*μ*mol/L, and 0.52 ± 0.12*μ*mol/L, respectively).

When comparing ADMA levels measured in umbilical blood of control newborns and newborns born to mothers with PE, Tsukahara et al. [84] found no significant difference between the two since their respective mean values were 1.71 ± 0.47*μ*mol/L and 1.66 ± 0.33*μ*mol/L. However, in a recent study by Gumus et al. [78], the median values for ADMA were noted to be significantly higher in umbilical blood from newborns born to mothers with PE than those from the control newborns (8.344ng/L versus 4.603ng/L). It was also noted that the level of ADMA measured from the umbilical cord blood sample correlated with the severity of the preeclamptic disorder.

#### 3.2.2. Methyl-Lysine

**Figure 13:**
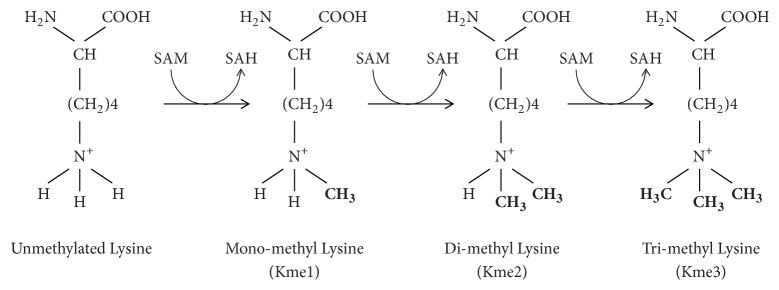
Formation of mono-, di-, and trimethyl-lysine.

In the case of lysine methylation, specific protein lysine methyltransferases (KMTs) catalyse the transfer of one, two, or three methyl groups from SAM to the epsilon (*ε*)-amine group of the side chain of a particular lysine residue [142]. This results in the formation of different forms of methylated lysines, namely, monomethyl-, dimethyl-, and trimethyl-lysines, respectively [142] (Figure 13). Some protein KMTs are specific for one or two of these modifications while others may result in the formation of all three derivatives [161]. Thus, it has been shown that these enzymes express product specificity since the type of methyl-lysine that is produced depends on the particular enzyme catalysing the reaction [162].

Nine functional members of the PRMT family have been identified (PRMT1-9) and the specificity of these enzymes for protein substrates varies and is generally much broader than that of KMTs. For instance, it has been shown that PRMT1, 2, 3, 4, 6, and 8 catalyse asymmetric dimethylation of arginine residues while enzyme PRMT5 catalyses symmetric dimethylation and PRMT7 may only catalyse monomethylation [163–165].

Most methyltransferase enzymes are grouped according to their structural features into three large families, namely, seven beta (*β*) strand [166], SET (suppressor of variegation 3-9 (Su(var)3-9), enhancer of zeste (E(z)), and trithorax (Trx)) domain-containing [167], and SPOUT domain-containing [168] enzymes. However, while all PRMTs belong to the seven *β* strand family of enzymes, most of the KMTs contain a conserved SET domain [169], which harbours the enzymatic activity of these proteins [170], and hence belong to the SET domain-containing family [171, 172]. Furthermore, an increasing number of enzymes which belong to the seven *β* strand family have been shown to catalyse similar methylation reactions [173–176]. Thus, KMTs can be broadly divided according to their enzymatic domain into SET domain-containing and non-SET domain-containing proteins.

The SET domain-containing KMTs have been classified into a number of families according to the sequence motifs surrounding the SET domain. Members of the same family share similar sequence motifs surrounding the SET domain and often also share a higher level of similarity in the SET domain. Seven main families are known and these include the suppressor of variegation (Su(var)) 3-9 (SUV39), SET1, SET2, enhancer of zeste (E(z)), retinoblastoma-interacting zinc-finger protein (RIZ), SET and Myeloid-Nervy-DEAF1 (MYND) domain-containing protein (SMYD), and suppressor of variegation (Su(var)) 4-20 (SUV4-20) families. These families are accompanied by SET7/9 and SET8 (also known as PR-SET7) which are SET domain-containing KMTs but do not fit in with the previously mentioned families [177]. A tabulated list of the KMTs found in humans which belong to each KMTs family, as well as SET7/9 and SET8, together with their properties has been presented by Dillon et al. [177].

Although a large majority of KMTs contain the SET domain, numerous other proteins which do not contain the SET domain, including the disruptor of telomeric silencing (DOT) 1-like (DOT1L) [169, 178] and methyltransferase-like (METTL) family proteins [179], also have lysine methyltransferase activity.

*(1) Lysine Methylation in Disease States and Pregnancy.* Along the years, numerous KMTs and lysine demethylases (KDMs) have been identified and their activity has been reported to be important in several biological processes, including the regulation of gene expression, cell-cycle progression, DNA replication, and differentiation [180–185]. In normal, healthy states, lysine methylation is tightly controlled and a balance in lysine methylation is maintained by the opposing actions of KMTs and KDMs [186]. At the same time, gene expression patterns must be able to respond to developmental requirements and environmental changes in order to maintain a healthy state [187].

The dysregulation of PTMs in the form of inappropriate expression (inclusion or elimination), as well as mutation of numerous KMTs and KDMs, may be a critical determinant of different diseases, including ageing and cancer [186–191]. In fact, the loss of this appropriate balance in methylation in adult stem cells has been thought to contribute to the decline of tissue function with age [192]. Studies have also shown that aberrant methylation is associated with an increased incidence of various types of cancers and poor survival [193, 194]. For instance, the methyltransferase responsible for histone 3 lysine 27 trimethylation (H3K27me3) is upregulated in prostate cancer [195], breast cancer [196], and lymphomas [197].

The human genome encodes over 200 methyltransferases [198] and although most studies have focused on histone methylation, a number of reports have revealed that these enzymes are also responsible for the regulation of methylation of nonhistone proteins [199]. It has been observed that a number of nonhistone proteins undergo methylation on their lysine residues and this in turn leads to changes in the function and/or stability of these nonhistone proteins [199, 200]. Evidence of lysine methylation-dependent regulation for an ever-increasing number of nonhistone proteins has been reported and in some cases these changes would also be of relevance to stress, hypertension, and PE as described below. 

*Lysine Methylation of p53*. The tumour suppressor protein p53 functions as a sequence-specific transcription factor which regulates important cellular processes including cell-cycle arrest, DNA repair, apoptosis, and senescence in response to stress signals. Under normal conditions, the level of p53 in the cell is maintained low; however, p53 is rapidly stabilised and activated in response to cellular stresses such as DNA damage and hypoxic states [143].

Trophoblast apoptosis in the human placenta has been shown to increase during the gestational period [201]. Furthermore, in pregnancies complicated by PE, dysregulation of cell turnover, which results in increased apoptosis [202–204] and reduced syncytiotrophoblast area [205], has been noted. The impact of exaggerated apoptosis on the placental pathology in cases of PE is unclear; however, this may ultimately prevent the replenishment of the syncytiotrophoblast, promote degeneration of the syncytium, and result in the release of vasoactive or inflammatory factors into the maternal circulation [206]. Since p53 is a vital regulator of the apoptotic pathway, its level has been measured in cases of PE and it has been observed that, at the protein level, the level of p53 is significantly elevated in placentas obtained from pregnancies complicated by PE [207]. This increase in p53 expression was also noted in cases of foetal IUGR [208, 209]. Also, the increase in p53 levels was associated with an increased expression of downstream elements of the apoptotic pathway, including the level of the downstream effector protein p21 [207].

**Figure 14:**
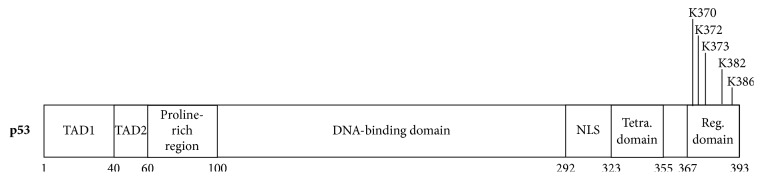
p53 lysine methylation sites (based on PhosphoSite data).

Methylation of p53 by SET7 (KMT7) was the first KMT-mediated methylation of a nonhistone protein reported [210]. Since then, a number of other KMTs, including SET9 (KMT5), SMYD2 (KMT3C), and SET 8 (KMT5A), which methylate p53 at specific C-terminal lysines, together with the lysine-specific demethylase KDM1(LSD1) which mediates p53 demethylation, have also been identified [143] (Figure 14).

P53 undergoes multiple PTMs, including lysine methylation, which regulate its stability, protein-protein interactions, and transcriptional activity. In fact, the transcriptional activity of p53 is enhanced or suppressed depending on the methylation site. Also, the interaction of p53 with its coactivator p53 binding protein 1 (53BP1) to induce apoptosis is mediated through the action of the lysine demethylase KDM1. The balance between methylation and demethylation, in combination with other PTMs, is essential in the response of p53 to cellular stresses since its activity is important in the prevention of tumour formation [143].

*Lysine Methylation of Heat Shock Protein (HSP) 70*. HSPs are primarily known as intracellular proteins that have molecular chaperone and cytoprotective functions [211] and are essential for cell recovery, survival, and maintenance of homeostasis [212]. HSP70 proteins are ubiquitous, adenosine triphosphate- (ATP-) dependent molecular chaperones which make up one of the most evolutionarily conserved family of proteins [213]. Extracellular HSP70 may contribute to the development of autoimmune disease and may provide an indication of the status of the innate immune system [214–216]. In humans, these proteins are encoded by 13 genes and they are either induced in response to stress (such as heat shock) or constitutively expressed [217]. HSP70 proteins carry out numerous biological processes including protein-protein interactions, protein degradation, and translocation across membranes [218].

HSP70 has been shown to be elevated in cases of PE [212, 219]. Park et al. [220] suggested that increased levels of systemic HSP70 in preeclamptic patients originate from syncytiotrophoblasts and villous endothelial cells of preeclamptic placentas since these were shown to have higher HSP70 proteins levels when compared to placentas from normal, healthy pregnancies. Other studies have also detected higher HSP70 levels in placental tissues of preeclamptic patients [221, 222]. This increase in serum HSP70 reflects systemic inflammation and oxidative stress that is noted in PE [212, 219]. Initially, expression of HSP70 plays a protective role against placental oxidative stress; however, the overexpression of HSP70 may lead to intervillous endothelial dysfunction and may play a role in the pathogenesis of PE [220].

**Figure 15:**
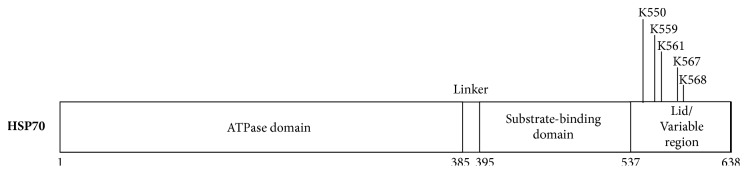
HSP70 lysine methylation sites (based on PhosphoSite data).

It has been shown that HSP70 can be posttranslationally modified in a number of ways, and numerous methylated lysines have been reported (Figure 15). For instance, it has been reported that the lysine residue K561, which is found in several human HSP70 proteins, can be methylated. Lysine methylation of HSP70 proteins is physiologically significant, especially in tumourigenesis. In particular, dimethylation of K561 of HSP70 by SETD1A regulates the subcellular localisation of this protein and it promotes the proliferation of cancer cells through its interaction with Aurora Kinase B (AURKB) [143, 223].

*Lysine Methylation of Vascular Endothelial Growth Factor Receptor 1 (VEGFR-1) and 2 (VEGFR-2).* As was described in previous sections, VEGFR-1 (or Flt-1) and VEGFR-2 are membrane receptors which belong to a receptor tyrosine kinase (RTK) subfamily. VEGRF-1 is able to bind tightly to its ligands, VEGF (including VEGF-A) and PlGF, but has weak tyrosine kinase activity and hence generates an overall weaker signal than other RTKs [224]. Through its interaction with its ligands, VEGFR-1 plays an important role in both physiologic and pathologic angiogenesis, the process by which new blood vessels are formed from preexisting vessels [43, 225, 226]. Angiogenesis is crucial for the normal physiological functions of tissues, but it is also important for the progression of certain diseases, including cancer and inflammation [227, 228].

VEGFR-1 has also been shown to be important in the regulation of vasculogenesis, which is the process by which new blood vessels are formed from precursor cells during early embryogenesis [225]. In 1995, Fong et al. [229] demonstrated that mutant mice which do not express VEGFR-1 (Flt-1-null, Flt-1-/- mice) die in utero due to the uncontrolled growth of vascular endothelial cells and disorganisation of blood vessels, which indicates that VEGFR-1 may have a negative regulatory role in angiogenesis during early embryogenesis.

VEGFR-1, together with its ligand VEGF and the other receptors for VEGF, is expressed throughout the gestational period in the placenta and these are essential for embryonic vascular development [34, 35]. During normal, uncomplicated pregnancies, VEGFR-1 can be primarily detected in placental syncytiotrophoblasts [230]. Altered levels of VEGF and its receptors during pregnancy can lead to a disruption in angiogenesis which results in placental insufficiency and endothelial dysfunction, both of which are noted in pregnancies complicated by PE [231]. In the study by Helske et al. [230], the expression of VEGFR-1 was seen to be increased in the syncytiotrophoblasts of placentas obtained from a number of cases of PE, but not in all.

**Figure 16:**
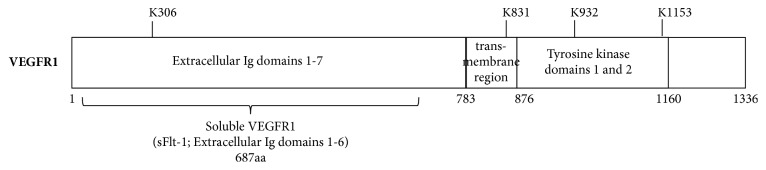
VEGFR1 lysine methylation sites (based on PhosphoSite data).

VEGFR-1 undergoes methylation at multiple lysine residues and it has been observed that this methylation is important in the regulation of the activity of VEGFR-1 (Figure 16). For instance, it has been shown that VEGFR-1 is a nonhistone target of SMYD3 methyltransferase since lysine 831 of VEGFR-1 is methylated by SMYD3 in vitro [232]. This methylation of VEGFR-1 enhances its kinase activity since lysine 831 is located within the kinase domain of this RTK.

VEGFR-2 is a potent angiogenic RTK, thus making it one of the most important RTKs in endothelial cells [233, 234]. The activity of VEGFR-2 has been noted to be essential in both vasculogenesis and pathological angiogenesis during cancer and ocular neurovascularisation [226].

Consequently, the expression and function of VEGFR-2 are highly regulated since increased angiogenesis plays a significant role in the progression of cancer and other diseases, including age-related macular degeneration, whereas insufficient angiogenesis has been linked to coronary heart disease and delayed wound healing [234]. VEGFR-2 undergoes methylation at multiple lysine residues and it has been shown that this methylation is important in the regulation of VEGFR-2 activity [233]. In fact, Hartsough et al. [233] also determined that methylation of lysine 1043 is essential in controlling the activation of VEGFR-2 since this methylation reaction is important for the tyrosine phosphorylation of this RTK. Consequently, interference of this methylation of VEGFR-2 results in an inability of VEGFR-2 to stimulate angiogenesis.

## 4. Conclusion

As with all biomarkers, their effectiveness is determined by the ability to diagnose the disease well before the presentation of clinical symptoms, and the advantage is that the changes can be detected through the minimally invasive blood tests, performed as part of the routine checks. So far, PlGF and sFlt-1, alone or in combination, not only have shown promise for the early diagnosis of PE but also have been shown to correlate well to the severity of this condition, with data from different cohorts being comparable. Similarly, the screening for ADMA has produced interesting trends and can be considered a useful candidate biomarker also based on the knowledge of its role in NO biochemistry. Our laboratory is also interested in exploring lysine methylation in conjunction with the above-mentioned potential biomarkers in order to extend the understanding of the role played by protein methylation in normal and PE biochemistry. Aside from the biomarkers selected, the final aim is to improve pregnancy progression and outcome, as well as to reduce the risks for both mother and foetus. This can only be achieved by unravelling and better understanding the underlying mechanisms leading to the preeclamptic condition.

## Figures and Tables

**Table 1:** Symptoms presented by patients with mild and severe PE. The diagnosis of any form of PE requires the presentation of both hypertension and proteinuria. This may be accompanied by a multitude of other symptoms if the PE is severe [8, 9].

**Symptom**	**Mild PE**	**Severe PE**
Blood Pressure	Systolic ≥140 mm Hg or diastolic ≥90 mm Hg, over 20 weeks of gestation (in a woman with previously normal blood pressure)	Systolic ≥160 mm Hg or diastolic ≥110 mm Hg (on two occasions at least six hours apart; in a woman on bed rest)

Proteinuria	24-hour urine collection protein ≥0.3 g (urine dipstick test ≥1+)	24-hour urine collection protein ≥5 g (urine dipstick test ≥3+; in two random urine samples collected at least four hours apart)

Others	N.A.	(i) Oliguria
		(ii) Cerebral or visual disturbances
		(iii) Pulmonary oedema or cyanosis
		(iv) Epigastric or right upper quadrant pain
		(v) Impaired liver function
		(vi) Thrombocytopenia
		(vii) Intrauterine growth restriction

**Table 2:** List of proposed serum biomarkers for the detection and diagnosis of PE.

**Proposed biomarker**	**Biological role**	**Serum level in PE compared to normotensive pregnancy**	**Type of study**	**Positive predictive value**	**References**
Soluble fms-like tyrosine kinase 1 (sFlt-1)	Anti-angiogenic factor	Higher	Case-control	No	[24, 46–51]
			Nested case-control	No	[52, 53]
			Cross-sectional case-control	No	[54–56]
			Longitudinal case-control	No	[57, 58]
			Prospective cohort	No	[59, 60]
				Yes	[61]
			Prospective nested case-control	No	[62]

Placental growth factor (PlGF)	Angiogenic factor	Lower	Case-control	No	[24, 46, 48, 50, 51]
				Yes	[63–65]
			Nested case-control	No	[52, 53, 66]
			Cross-sectional case-control	No	[54–56]
			Longitudinal case-control	No	[57, 58, 67]
				Yes	[68]
			Prospective cohort	No	[59, 60]
				Yes	[61]
			Prospective nested case-control	No	[62]
			Prospective longitudinal case-control	No	[69]
			Longitudinal cohort	No	[70]
			Longitudinal cross-sectional	No	[71, 72]

Asymmetric Dimethyl-Arginine (ADMA)	Biochemical degradation product	Higher	Case-control	No	[73–77]
			Longitudinal case-control	No	[78–82]
			Cross-sectional case-control	No	[83, 84]

Soluble Endoglin (sEng)	Modulator of transforming growth factor (TGF)-*β* signalling	Higher	Longitudinal case-control	No	[58]
			Cross-sectional case-control	No	[85, 86]
			Nested case-control	No	[87, 88]
			Retrospective	No	[89]
			Prospective	No	[90]
			Case-control	No	[91]

Placental Protein 13 (PP-13)	Lysophospholipase activity	Higher	Longitudinal case-control	No	[92, 93]
			Nested case-control	No	[94–98]
			Case-control	No	[99]

P-Selectin	Calcium-dependent receptor	Higher	Cross-sectional case-control	No	[100–105]
				Yes	[106]
			Longitudinal case-control	No	[107]
				Yes	[108]

Adrenomedullin	Vasodilator	Higher	Cross-sectional case-control	No	[109]

A Disintegrin and Metalloprotease 12 (ADAM12)	Cell-cell and cell-matrix interaction protease	Lower	Retrospective case-control	No	[110]
			Cross-sectional case-control	No	[111, 112]

Pentraxin 3 (PTX3)	Angiogenesis and inflammation factor	Higher	Cross-sectional case-control	No	[113, 114]

Pregnancy-Associated Plasma Protein A (PAPP-A)	Metalloproteinase that cleaves insulin-like growth factor binding proteins (IGFBPs)	Lower	Case-control	Yes	[64]
			Nested case-control	No	[98]
			Cross-sectional case-control	No	[115–118]
			Retrospective cohort	No	[119–122]
			Prospective cohort	No	[123]

Nicotinamide Phosphoribosyltransferase; Visfatin	Enzyme involved in nicotinamide metabolism	Both	Cross-sectional case-control	No	[124]

Cell free DNA	N.A	Higher	Cross-sectional case-control	No	[125–127]

Cell-free foetal DNA	N.A.	Higher	Cross-sectional case-control	No	[127–129]
			Nested case-control	No	[130, 131]
			Prospective	No	[132, 133]
